# The Role of Microvesicles Derived from Mesenchymal Stem Cells in Lung Diseases

**DOI:** 10.1155/2015/985814

**Published:** 2015-05-12

**Authors:** Jie Chen, Chonghui Li, Liangan Chen

**Affiliations:** ^1^Department of Respiratory Medicine, Chinese People's Liberation Army General Hospital, Chinese People's Liberation Army Medical College, Beijing, China; ^2^Department of Hepatobiliary Surgery, Chinese PLA General Hospital, Chinese PLA Medical College, Beijing, China; ^3^Institute of Hepatobiliary Surgery, Chinese PLA General Hospital, Chinese PLA Medical College, Beijing, China

## Abstract

Microvesicles (MVs) are membrane vesicles that are released by many types of cells and have recently been considered important mediators of cell-to-cell communication. MVs serve as a vehicle to transfer proteins and messenger RNA and microRNA (miRNA) to distant cells, which alters the gene expression, proliferation, and differentiation of the recipient cells. Several studies have demonstrated that mesenchymal stem cells (MSCs) have the capacity to reverse acute and chronic lung injury in different experimental models through paracrine mechanisms. This paracrine action may be partially accounted for by MVs that are derived from MSCs. MSC-derived MVs may confer a stem cell-like phenotype to injured cells with the consequent activation of self-regenerative programmers. In this review, we summarize the characteristics and biological activities of MSC-derived MVs, and we describe their potential in novel therapeutic approaches in regenerative medicine to repair damaged tissues. Additionally, we provide an overview of studies that have assessed the role of MSC-derived MVs in lung diseases, including the mechanisms that may account for their therapeutic potential. Finally, we discuss the clinical use of MSC-derived MVs with several suggestions for enhancing their therapeutic efficiency.

## 1. Introduction

MSCs, also known as mesenchymal stromal cells, were originally identified in bone marrow [1]. To date, MSCs can be isolated from a wide variety of other tissues, including umbilical cord blood, Wharton's jelly, and placental, adipose, and lung tissue [2]. According to the definition by the International Society for Cellular Therapy, MSCs are generally evaluated according to the following three main biological criteria [3]: plastic adherence when maintained in standard culture conditions, the expression of CD105, CD73, and CD90 and no expression of CD45, CD34, CD14, CD11b, CD79a, CD19, or HLA-DR surface molecules, and differentiation to osteoblasts, adipocytes, and chondroblasts* in vitro*. Over the last decade, MSCs have emerged as an attractive cell-based therapeutic candidate in lung injury because of their ability to migrate to the site of injury, their potential to differentiate to lung epithelium cells, and their straightforward* in vitro* expansion [4]. Because of these clinically useful features, MSCs have provoked enthusiasm for their application in lung injury.

Recently, growing evidence has indicated that the beneficial effect of MSCs in lung diseases is not attributed to their differentiation capacity but rather to the activation of a protective mechanism and the stimulation of endogenous regeneration [5–7]. This conclusion is drawn from the observation that MSCs can produce bioactive soluble factors that are known to reduce the permeability of the alveolocapillary membrane, inhibit apoptosis and fibrosis, decrease inflammation, and enhance tissue repair [5, 8–10]. MSC-secreted bioactive molecules may act as paracrine or endocrine mediators that interact with neighboring cells, modulate immune responses, and promote self-repair from cells that survive injury [11–13]. In this context, microvesicles (MVs) that are released from stem cells may account for a reciprocal communication between stem and injured tissue cells.

## 2. The Paracrine/Endocrine Effect of MSCs in Lung Regeneration

The role of MSCs in recovery from lung injury has been extensively studied. MSCs have been demonstrated to accelerate recovery from lung injury induced by endotoxin [6, 8, 9], bleomycin [10], and radiation [14]. In addition, MSCs have induced functional improvement in ventilator-induced lung injury [15]. Early evidence has indicated that systemically administered MSCs could be retained in the recipient lung, but few MSCs could be permanently engrafted within the lung [5, 6]. This observation suggests that the beneficial effects of MSCs' infusion in lung injury are not dependent on a direct substitution of injured cells but rather on paracrine effectors that facilitate endogenous repair processes. Qin et al. [12] demonstrated that the intrapleural delivery of MSCs could markedly attenuate the severity of endotoxin-induced ALI in rats. This study demonstrated that the paracrine/endocrine mechanism of MSCs played a role in the repair of ALI because no evidence of MSC engraftment to lung tissue was found. Additionally, in a mouse model of LPS-induced lung injury, MSC-conditioned medium (MSC-CM) mimicked the beneficial effects of the cells of origin and promoted the resolution of lung injury by attenuating lung inflammation [11], which further supports the paracrine/endocrine action of MSCs. Moreover, Lee and colleagues [8] demonstrated that treatment with human MSC-CM reduced extravascular lung water, improved lung endothelial barrier permeability, and restored alveolar fluid clearance in an* ex vivo* perfused human lung that was injured by endotoxin. Several studies have demonstrated that the protective effect of MSCs in ALI depends on secreted factors [7, 8, 11, 13, 16], such as keratinocyte growth factor (KGF) [8, 13], angiopoietin-1 (Ang1) [16], and insulin-like growth factor (IGF-I) [11]. KGF gene and Ang1 gene silencing limited the protective effect of MSCs on lung endothelial permeability, and recombinant IGF-I partially reproduced the protective effect of MSC-CM on lungs in ALI. Recently, several studies found that MSCs have antimicrobial effects through the soluble factor antimicrobial peptide LL-37 [17].

The paracrine action of MSCs is associated with their immunomodulatory properties. Multiple studies have found that MSCs possess potent immunosuppressive effects by inhibiting the activity of both innate and adaptive immune cells [11, 18]. This immunosuppression is mediated by cell-to-cell interactions and the secretion of soluble factors, including members of the transforming growth factor-*β* (TGF-*β*) family, interleukin-10 (IL10), interleukin-1RA (IL-1RA), nitric oxide, and indoleamine 2,3 dioxygenase (IDO) [18]. MSCs can alter the cytokine production of dendritic cells (DCs), naïve and effector T-cells, and natural killer (NK) cells, which results in a more tolerant, anti-inflammatory phenotype. Moreover, different studies have demonstrated that MSCs suppressed T-cell proliferation, most likely via prostaglandin E2 (PGE2) production [19]. Despite the well-documented immunosuppressive effects of MSCs, recent evidence has indicated that MSCs can act as immunostimulatory cells. Several studies have reported that MSCs can upregulate the expression of MHC II when exposed to low levels of inflammation and that MSCs function as antigen-presenting cells to stimulate the adaptive immune system [20, 21]. In addition, evidence suggests that MSCs can secrete IL-6 and induce the production of IgG by B lymphocytes in an* in vitro* setting [18]. In addition, Sun and colleagues [22] reported that the transplantation of MSCs ameliorated ALI in mice by enhancing diminished levels of alveolar CD4+ CD25+ Foxp3+ Treg and by balancing anti- and proinflammatory factors.

## 3. The Formation and Main Characteristics of Microvesicles

In addition to soluble factors, recent studies have demonstrated that small vesicles that are released from cells, named extracellular vesicles (EVs), are instrumental in cell-to-cell communication [23, 24]. EVs are a group of small vesicles that are constituted by a circular fragment of membrane that contains cytoplasm components. EVs are released by different cell types. The two major classes of EVs that are released in the extracellular environment are exosomes and shedding vesicles. Exosomes arise from the endosomal membrane cell compartment and are released into the extracellular space after the fusion of multivesicular bodies with the plasma membrane [3, 24]. Exosomes have a diameter of 40–100 nm, a homogeneous shape, and a density of 1.13–1.19 g/mL in sucrose. The release of exosomes is dependent on cytoskeleton activation but not on Ca2+ influx. Exosomes have an evolutionarily conserved set of proteins, including tetraspanins (CD63, CD81, and CD9), heat-shock proteins (Hsp60, Hsp70, and Hsp90), tumor susceptibility gene 101 (Tsg101), Alix, clathrin, and annexins [3, 24]. Shedding vesicles, also named ectosomes or membrane particles, are more heterogeneous in size and include vesicles that range from 100 nm to 1 *μ*m in size. Shedding vesicles originate from the direct budding of small cytoplasmic protrusions, followed by their detachment from the cell surface [3, 24, 25]. This process is dependent on cytoskeleton activation and an increase in intracellular calcium concentrations, which modify the asymmetric phospholipid distribution of plasma membranes by specific enzymes named calpain, flippase, floppase, scramblase, and gelsolin. Shedding vesicles expose large amounts of phosphatidylserine and are enriched in proteins that are associated with membrane lipid rafts [3, 24, 25]. Both exosomes and shedding vesicles are present* in vitro* and* in vivo*; therefore, this mixed population is collectively known as microvesicles (MVs). MVs contain surface receptors, biologically active molecules, such as proteins and lipids, and mRNA and microRNA, which play a role in the exchange of genetic material between cells. Apoptotic bodies represent another type of EV, which are larger than exosomes and shedding vesicles (1–5 mm in diameter) and are released from the plasma membrane as blebs when cells undergo apoptosis. Apoptotic bodies are characterized by phosphatidylserine externalization and contain several intracellular fragments and cellular organelles, including histones and fragmented DNA [3, 24, 25].

## 4. The Biological Activities of Microvesicles

MVs retain the signature of the cell of origin. They contain proteins (e.g., receptors and adhesion molecules), lipids of the cell membrane, and cytoplasmic constituents [mRNA, microRNA (miRNA), DNA, and proteins]. The release of MVs may be constitutive or consequent to cell activation by soluble agonists, physical or chemical stress, such as oxidative stress and hypoxia, and shear stress [26]. This process is an evolutionarily well-conserved mechanism that cells exploit for the exchange of bioactive proteins, lipids, and nucleic acids [25].

**Figure 1:**
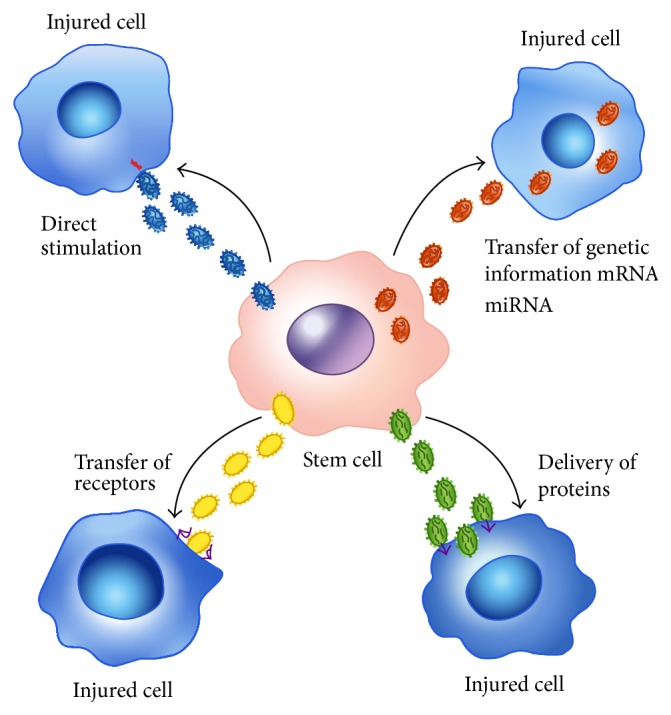
A schematic representation of the mechanisms involved in the exchange of information between stem cells and injured tissue cells as mediated by microvesicles. (a) MVs may directly stimulate target cells through surface-expressed receptors. (b) MVs may transfer receptors to target cells. (c) MVs may deliver functional proteins to target cells. (d) MVs may transfer genetic information via mRNA and microRNA (miRNA) to target cells.

MVs can function as cargo for delivering cellular components to other cells, thereby inducing alterations in the phenotype and behavior of recipient cells [25]. MVs influence the behavior of recipient cells in multiple ways (Figure 1).

First, MVs may act as signaling complexes by the direct stimulation of target cells. Several studies have demonstrated that MVs express several types of receptors and surface molecules, including tissue factor (TF), tumor necrosis factor (TNF), MHC class I/II molecules, and the CCR5 chemokine receptor [3, 24]. This expression results in MV-mediated activation of cells that bear specific ligands for these receptors. MVs that are derived from platelets have an important role in coagulation because their phosphatidylserine-enriched membranes provide a surface for the assembly of clotting factors. After activation, platelet-derived MVs are coated with tissue factor, which may interact with macrophages, neutrophils, and other platelets during the ligation of molecules that are expressed on the surface of these cells, such as P-selectin [23].

MVs may transfer receptors and/or bioactive lipids between cells. MVs can transfer the adhesion molecule CD41 from platelets to endothelial cells [27] or tumor cells [28], thereby conferring proadhesive properties to these cells. In addition, MVs may contribute to spreading certain infective agents, such as human immunodeficiency virus type 1. The transfer of the CXC chemokine receptor 4 (CXCR4) and CC chemokine receptor 5 (CCR5) coreceptors for the human immunodeficiency virus type 1 by MVs may favor virus entry into cells of lineages over the lymphohemtopoietic lineage [3, 29].

MVs may modulate functional target cells by delivering intracellular proteins. MVs that are derived from endothelial cells can activate angiogenesis through the transfer of proangiogenic molecules, such as growth factors (e.g., vascular endothelial growth factor, VEGF; basic fibroblast growth factor, bFGF; platelet-derived growth factor, PDGF; and TGF-*β*) [3, 30], proteases [e.g., matrix metalloproteinase 9 (MMP9), MMP2, and membrane-type 1 MMP (MT1-MMP)] [3, 31] and their activator, and extracellular MMP inducer (EMMPRIN) [32]. Endotoxin-stimulated monocytes induce the cell death of vascular smooth muscle cells by releasing MVs that contain caspase-1 [3, 33]. This induction of the transcellular apoptosis pathway depends on the function of caspase-1 within target cells.

Recently, it has been demonstrated that MVs may mediate the horizontal transfer of genetic information. Ratajczak et al. [34] demonstrated that MVs that were derived from human endothelial progenitor cells (EPCs) shuttle mRNA to endothelial cells via an interaction with *α*4- and *β*1-integrins that are expressed on their surface, thereby activating an angiogenic program. The molecular analysis of mRNA indicated that MVs that were derived from endothelial progenitor cells were shuttling a specific subset of cellular mRNA, including mRNA that is associated with pathways for angiogenesis, such as the PI3K/AKT and endothelial nitric oxide synthase signaling pathways [23, 35]. The pretreatment of MVs with RNase abrogated the angiogenic effect despite MV internalization by endothelial cells, which confirms the critical role of MVs in RNA transfer [35]. Moreover, MVs that are released by bone marrow MSCs (BM-MSCs) contain mRNA for the insulin growth factor 1 (IGF-1) receptor [36]. In an* in vitro* model of renal toxic injury induced by cisplatin, the transfer of IGF-1 receptor mRNA through MVs increased the proliferation of damaged proximal tubular cells [36]. In addition, MVs that are released from human MSCs and human liver stem cells (HLSCs) contain ribonucleoproteins that are involved in the intracellular trafficking of RNA and selected patterns of microRNA, which suggests the dynamic regulation and compartmentalization of RNA in MVs that are produced by human adult stem cells of mesenchymal origin [3, 37]. Because miRNAs are naturally occurring regulators of protein translation, this observation suggests that stem cells can alter the expression of gene products in neighboring cells by transferring miRNAs in MVs [37]. Based on a Gene Ontology analysis, the predicted and validated targets of the miRNAs in MSC-MVs are related to cell development, survival, and differentiation, whereas several MSC MV-enriched miRNAs were associated with the regulation of the immune system [37].

MVs released from MSCs may be considered potent paracrine/endocrine factors that are involved in the signaling between stem cells and differentiated cells. Dooner et al. [38] demonstrated that bone marrow cells that were cocultured with injured lung cells expressed genes for lung-specific proteins, such as surfactant B and C, and Clara cell-specific proteins. This phenomenon may be due to the transfer of lung-specific mRNAs to bone marrow cells via MVs that were released from the injured lung cells. Therefore, stem cells and differentiated cells may establish bidirectional communication during the reparative process [38]. MVs that are released from injured cells may deliver specific signals to stem cells that may trigger their differentiation and may represent a mechanism of physiological tissue repair [23]. In contrast, stem cell-derived MVs may incite changes in the phenotype of tissue cells that regulate cell regeneration and differentiation.

## 5. The Therapeutic Potential of MSC-Derived MVs in Lung Regeneration

The regenerative potential of MSC-derived MVs has been demonstrated in different animal models of lung tissue injury. Lee et al. demonstrated that exosomes mediate the paracrine anti-inflammatory effect of bone marrow MSCs during hypoxia-induced pulmonary hypertension [39]. In this study, MSC-derived exosomes were identified through widely accepted exosomal markers and visualized by electron microscopy. The intravenous delivery of MSC-derived exosomes protected against the elevation of right ventricular systolic pressure and the development of RVH after three weeks of hypoxic exposure, whereas MV-depleted media or fibroblast-derived exosomes had no effect. In addition, the exosomal treatment abrogated early hypoxic macrophage influx and downregulated hypoxia-activated inflammatory pathways, thereby mediating the anti-inflammatory properties of MSCs. Exosome action was further validated* in vitro*: exosomes ameliorated the hypoxic induction of STAT3 in pulmonary artery endothelial cells and increased miR-204 levels in the lungs [39]. Additionally, MVs that were derived from MSCs were therapeutically effective following E. coli endotoxin-induced ALI in mice in part through the expression of KGF mRNA in the injured alveolus. In this model, MVs were intratracheally instilled 48 hours after endotoxin administration. MVs reduced extravascular lung water by 43% and the total protein levels in bronchoalveolar lavage (BAL) fluid by 35% when the MV-treated mice were compared with the control mice. Therefore, the administration of MVs led to a reduction in pulmonary edema and lung protein permeability. Additionally, MVs reduced neutrophil influx and macrophage inflammatory protein-2 levels in BAL fluid by 73% and 49%, respectively. Therefore, the administration of MVs led to a reduction in inflammation. However, the therapeutic effects were partially eliminated after the delivery of MVs that were derived from KGF siRNA-pretreated MSCs, which suggests that KGF protein expression was an important underlying mechanism [40].

A similar protective effect of MSC-derived MVs was observed in a model of acute kidney injury (AKI) induced by glycerol [41] or cisplatin [42]. In this model, MVs ameliorated renal function and morphology and improved survival. In another study, Xin et al. demonstrated that exosomes from MSCs mediated miR-133b transfer to astrocytes and neurons which regulate gene expression. This transfer subsequently benefited neurite remodeling and functional recovery after stroke [43]. In addition, MVs have been proposed as an alternative therapeutic vehicle for MSCs in a myocardial injury/reperfusion model, in which MSC exosomes decreased infarct size and ameliorated reperfusion injury [44]. Additionally, phase I trials of microvesicles derived from dendritic cells as immunotherapy for patients with advanced cancer revealed that the large-scale, clinical-grade production and delivery of MSC exosomes/MVs are feasible and safe.

## 6. Conclusion

Several studies have suggested a paracrine/endocrine mechanism in stem cell-mediated lung repair [6, 8, 12, 13]. However, the nature of the factors that are responsible for the beneficial paracrine/endocrine effect of stem cells has remained elusive. Recent work by several groups has suggested that MVs strongly contribute to the paracrine effects of stem cells [3, 23–26]. MVs have a complex composition that mirrors that of parental cells, and MVs have similar properties* in vivo*.

MVs demonstrate several possible advantages over stem cells in terms of their use in regenerative medicine. Importantly, MVs possess an intrinsic homing ability relative to other synthetic particles, which prevents their unwanted accumulation in organs other than the target tissue. MVs demonstrate no inherent toxicity and are not associated with any long-term maldifferentiation of engrafted cells, tumor generation, or immune rejection following the* in vivo* allogeneic administration of MVs [3]. Additionally, MVs may be engineered to express and deliver molecules that favor the reprogramming of resident cells toward regeneration.

Nevertheless, several problems need to be addressed before the clinical use of MVs may be considered. First, the potency of different MV preparations needs to be precisely defined. Second, strategies to obtain sufficient amounts of MVs need to be developed. A preliminary kinetic study based on the NanoSight detection of MVs in MSC-conditioned medium revealed that the timing of collection is critical. In addition, the enhanced release of MVs may be obtained after the appropriate stimulation of MSCs. Third, the exact mechanisms of the interaction between MVs and injured tissues and the homing and biodistribution of MVs require further investigation. Finally, further studies are needed to define the biosafety, fields of application, and effective doses of MSC-derived MVs in regenerative medicine. Recognizing the importance of MVs may lead to new perspectives in their investigation.

## References

[1] Friedenstein A. J., Petrakova K. V., Kurolesova A. I., Frolova G. P. (1968). Heterotopic of bone marrow. Analysis of precursor cells for osteogenic and hematopoietic tissues. *Transplantation*.

[2] Wang Y.-Y., Li X.-Z., Wang L.-B. (2013). Therapeutic implications of mesenchymal stem cells in acute lung injury/acute respiratory distress syndrome. *Stem Cell Research and Therapy*.

[3] Biancone L., Bruno S., Deregibus M. C., Tetta C., Camussi G. (2012). Therapeutic potential of mesenchymal stem cell-derived microvesicles. *Nephrology Dialysis Transplantation*.

[4] Baglio S. R., Pegtel D. M., Baldini N. (2012). Mesenchymal stem cell secreted vesicles provide novel opportunities in (stem) cell-free therapy. *Frontiers in Physiology*.

[5] van Haaften T., Byrne R., Bonnet S. (2009). Airway delivery of mesenchymal stem cells prevents arrested alveolar growth in neonatal lung injury in rats. *American Journal of Respiratory and Critical Care Medicine*.

[6] Yang K.-Y., Shih H.-C., How C.-K. (2011). IV delivery of induced pluripotent stem cells attenuates endotoxin-induced acute lung injury in mice. *Chest*.

[7] Lee J. W., Fang X., Krasnodembskaya A., Howard J. P., Matthay M. A. (2011). Concise review: mesenchymal stem cells for acute lung injury: role of paracrine soluble factors. *Stem Cells*.

[8] Lee J. W., Fang X., Gupta N., Serikov V., Matthay M. A. (2009). Allogeneic human mesenchymal stem cells for treatment of E. coli endotoxin-induced acute lung injury in the ex vivo perfused human lung. *Proceedings of the National Academy of Sciences of the United States of America*.

[9] Sun J., Han Z.-B., Liao W. (2011). Intrapulmonary delivery of human umbilical cord mesenchymal stem cells attenuates acute lung injury by expanding CD4^+^CD25^+^ forkhead Boxp3 (FOXP3)^+^ regulatory T cells and balancing anti- and pro-inflammatory factors. *Cellular Physiology and Biochemistry*.

[10] Jun D., Garat C., West J. (2011). The pathology of bleomycin-induced fibrosis is associated with loss of resident lung mesenchymal stem cells that regulate effector T-cell proliferation. *Stem Cells*.

[11] Ionescu L., Byrne R. N., van Haaften T. (2012). Stem cell conditioned medium improves acute lung injury in mice: in vivo evidence for stem cell paracrine action. *American Journal of Physiology: Lung Cellular and Molecular Physiology*.

[12] Qin Z.-H., Xu J.-F., Qu J.-M. (2012). Intrapleural delivery of MSCs attenuates acute lung injury by paracrine/endocrine mechanism. *Journal of Cellular and Molecular Medicine*.

[13] Chen J., Li C., Gao X. (2013). Keratinocyte growth factor gene delivery via mesenchymal stem cells protects against lipopolysaccharide-induced acute lung injury in mice. *PLoS ONE*.

[14] Wang H., Yang Y.-F., Zhao L. (2013). Hepatocyte growth factor gene-modified mesenchymal stem cells reduce radiation-induced lung injury. *Human Gene Therapy*.

[15] Curley G. F., Hayes M., Ansari B. (2012). Mesenchymal stem cells enhance recovery and repair following ventilator-induced lung injury in the rat. *Thorax*.

[16] Mei S. H. J., McCarter S. D., Deng Y., Parker C. H., Liles W. C., Stewart D. J. (2007). Prevention of LPS-induced acute lung injury in mice by mesenchymal stem cells overexpressing angiopoietin. *PLoS Medicine*.

[17] Krasnodembskaya A., Song Y., Fang X. (2010). Antibacterial effect of human mesenchymal stem cells is mediated in part from secretion of the antimicrobial peptide LL-37. *Stem Cells*.

[18] Lee J. W., Gupta N., Serikov V., Matthay M. A. (2009). Potential application of mesenchymal stem cells in acute lung injury. *Expert Opinion on Biological Therapy*.

[19] Aggarwal S., Pittenger M. F. (2005). Human mesenchymal stem cells modulate allogeneic immune cell responses. *Blood*.

[20] Chan J. L., Tang K. C., Patel A. P. (2006). Antigen-presenting property of mesenchymal stem cells occurs during a narrow window at low levels of interferon-*γ*. *Blood*.

[21] Stagg J., Pommey S., Eliopoulos N., Galipeau J. (2006). Interferon-*γ*-stimulated marrow stromal cells: a new type of nonhematopoietic antigen-presenting cell. *Blood*.

[22] Sun J., Han Z.-B., Liao W. (2011). Intrapulmonary delivery of human umbilical cord mesenchymal stem cells attenuates acute lung injury by expanding CD4^+^CD25^+^ forkhead Boxp3 (FOXP3) ^+^ regulatory T cells and balancing anti- and pro-inflammatory factors. *Cellular Physiology and Biochemistry*.

[23] Camussi G., Deregibus M. C., Bruno S., Cantaluppi V., Biancone L. (2010). Exosomes/microvesicles as a mechanism of cell-to-cell communication. *Kidney International*.

[24] György B., Szabó T. G., Pásztói M. (2011). Membrane vesicles, current state-of-the-art: emerging role of extracellular vesicles. *Cellular and Molecular Life Sciences*.

[25] Camussi G., Deregibus M. C., Cantaluppi V. (2013). Role of stem-cell-derived microvesicles in the paracrine action of stem cells. *Biochemical Society Transactions*.

[26] Bruno S., Camussi G. (2013). Role of mesenchymal stem cell-derived microvesicles in tissue repair. *Pediatric Nephrology*.

[27] Janowska-Wieczorek A., Majka M., Kijowski J. (2001). Platelet-derived microparticles bind to hematopoietic stem/progenitor cells and enhance their engraftment. *Blood*.

[28] Barry O. P., Praticò D., Savani R. C., FitzGerald G. A. (1998). Modulation of monocyte-endothelial cell interactions by platelet microparticles. *Journal of Clinical Investigation*.

[29] Mause S. F., Weber C. (2010). Microparticles: protagonists of a novel communication network for intercellular information exchange. *Circulation Research*.

[30] Taraboletti G., D'Ascenzo S., Giusti I. (2006). Bioavailability of VEGF in tumor-shed vesicles depends on vesicle burst induced by acidic pH 1. *Neoplasia*.

[31] Taraboletti G., D’Ascenzo S., Borsotti P., Giavazzi R., Pavan A., Dolo V. (2002). Shedding of the matrix metalloproteinases MMP-2, MMP-9, and MT1-MMP as membrane vesicle-associated components by endothelial cells. *The American Journal of Pathology*.

[32] Sidhu S. S., Mengistab A. T., Tauscher A. N., LaVail J., Basbaum C. (2004). The microvesicle as a vehicle for EMMPRin in tumor-stromal interactions. *Oncogene*.

[33] Sarkar A., Mitra S., Mehta S., Raices R., Wewers M. D. (2009). Monocyte derived microvesicles deliver a cell death message via encapsulated caspase-1. *PLoS ONE*.

[34] Ratajczak J., Miekus K., Kucia M. (2006). Embryonic stem cell-derived microvesicles reprogram hematopoietic progenitors: evidence for horizontal transfer of mRNA and protein delivery. *Leukemia*.

[35] Deregibus M. C., Cantaluppi V., Calogero R. (2007). Endothelial progenitor cell—derived microvesicles activate an angiogenic program in endothelial cells by a horizontal transfer of mRNA. *Blood*.

[36] Tomasoni S., Longaretti L., Rota C. (2013). Transfer of growth factor receptor mRNA via exosomes unravels the regenerative effect of mesenchymal stem cells. *Stem Cells and Development*.

[37] Collino F., Deregibus M. C., Bruno S. (2010). Microvesicles derived from adult human bone marrow and tissue specific mesenchymal stem cells shuttle selected pattern of miRNAs. *PLoS ONE*.

[38] Dooner M. S., Aliotta J. M., Pimentel J. (2008). Conversion potential of marrow cells into lung cells fluctuates with cytokine-induced cell cycle. *Stem Cells and Development*.

[39] Lee C., Mitsialis S. A., Aslam M. (2012). Exosomes mediate the cytoprotective action of mesenchymal stromal cells on hypoxia-induced pulmonary hypertension. *Circulation*.

[40] Zhu Y. G., Feng X. M., Abbott J. (2014). Human mesenchymal stem cell microvesicles for treatment of Escherichia coli endotoxin-induced acute lung injury in mice. *Stem Cells*.

[41] Bruno S., Grange C., Deregibus M. C. (2009). Mesenchymal stem cell-derived microvesicles protect against acute tubular injury. *Journal of the American Society of Nephrology*.

[42] Bruno S., Grange C., Collino F. (2012). Microvesicles derived from mesenchymal stem cells enhance survival in a lethal model of acute kidney injury. *PLoS ONE*.

[43] Xin H., Li Y., Liu Z. (2013). MiR-133b promotes neural plasticity and functional recovery after treatment of stroke with multipotent mesenchymal stromal cells in rats via transfer of exosome-enriched extracellular particles. *Stem Cells*.

[44] Lai R. C., Arslan F., Lee M. M. (2010). Exosome secreted by MSC reduces myocardial ischemia/reperfusion injury. *Stem Cell Research*.

